# Modification in structural, physicochemical, functional, and *in vitro* digestive properties of kiwi starch by high-power ultrasound treatment

**DOI:** 10.1016/j.ultsonch.2022.106004

**Published:** 2022-04-12

**Authors:** Jiaqi Wang, Xinran Lv, Tian Lan, Yushan Lei, Jiangtao Suo, Qinyu Zhao, Jing Lei, Xiangyu Sun, Tingting Ma

**Affiliations:** a College of Food Science and Engineering, College of Enology, Shaanxi Provincial Key Laboratory of Viti-Viniculture, Viti-viniculture Engineering Technology Center of State Forestry and Grassland Administration, Shaanxi Engineering Research Center for Viti-Viniculture, Heyang Viti-viniculture Station, Ningxia Eastern Foot of Helan Mountain Wine Station, Northwest A&F University, Yangling 712100, China; b Shaanxi Rural Science and Technology Development Center, Xi’an 710054, China; c Shaanxi Bairui Kiwifruit Research Co, Ltd., Xi’an 710054, China

**Keywords:** TPTZ, 2,4,6-tripyridine-s-triazine, R_1047/1022_, amplitude ratio of 1047/1022 cm^−1^, AAC, apparent amylose content, BD, breakdown value, CPV, cold paste viscosity, Tc, conclusion temperature, K, consistency coefficient, DSC, differential scanning calorimetry, DW, dry weight, ΔH, enthalpy change, FRAP, ferric reducing antioxidant power, n, fluid index, FTIR, fourier transform infrared spectroscopy, GAE, gallic acid equivalents, HUT, high-power ultrasound treatment, HPV, hot paste viscosity, KS, kiwi starch, G'', loss modulus, OAC, oil absorption capacity, To, onset temperature, PSD, Particle size distribution, PT, pasting temperature, Tp, peak temperature, PV, peak viscosity, PLM, Polarizing microscope, RVA, Rapid Visco-Analyzer, RDS, rapidly digestible starch, RC, relative crystallinity, RS, resistant starch, RS3, retrograded starch, SEM, Scanning electron microscopy, SB, setback value, SDS, slowly digestible starch, SD, standard deviations, G', storage modulus, SP, swelling power, Ptime, time to peak viscosity, TPC, total polyphenol content, TE, trolox equivalents, UST, ultrasonic treatment, WSI, water solubility index, XRD, X-ray diffraction, Kiwi starch, Modified starch, High-power ultrasound, Characterization, Digestive properties, Alternative starch resource

## Abstract

Kiwi starch (KS) is a fruit-derived starch; in order to improve its processing performance and increase its added value, it is necessary to modify KS to enhance the positive attributes and to enlarge its application. In this study, KS was modified by high-power ultrasound treatment (HUT) to reveal the relationship between the structure and function of KS with different treatment powers (0, 200, 400, and 600 W) and different treatment times (0, 10, 20, and 30 min). The results showed that HUT destroyed the granular morphology of KS, formed holes and cracks on the surface, and reduced the particle size and the short-range molecular order of KS. After different HUTs, the apparent amylose content (AAC), swelling power (SP), water solubility index (WSI), viscosity and setback value (SB) of KS were significantly increased, while the gelatinization temperature was significantly decreased. In addition, HUT significantly reduced the content of rapidly digestible starch (RDS) and slowly digestible starch (SDS), while it significantly enhanced the content of resistant starch (RS) (64.08–72.73%). In a word, HUT as a novel physical modification method for KS, enlarged its application, and fulfilled different demands of a starch-based product, which introduces another possibility for kiwi fruit further processing.

## Highlights

• Kiwi starch (KS) was modified by high-power ultrasound treatment (HUT).
• HUT enhanced the swelling power, solubility, and oil absorption capacity of KS.
• Destruction of surface, decrease in R_1047/1022_, and crystallinity was found in KS.
• HUT markedly increased the content of apparent amylose and resistant starch in KS.
• The relationship between the structures and properties of KS was revealed.

## Graphical abstract

**Figure:**
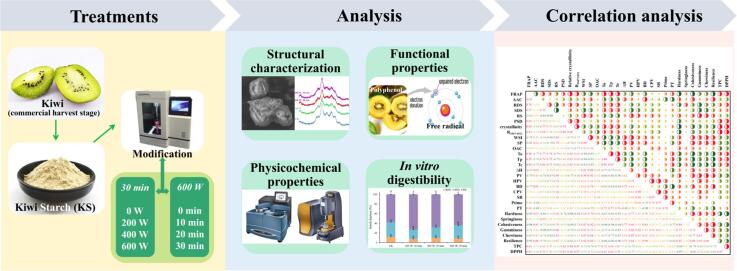

## 1 Introduction

Starch is the main dietary source of carbohydrates and the most abundant storage polysaccharide in plants, which exists in the amyloplast of plant fruits, pericarps, or seeds [1]. They can be obtained from a variety of plant sources such as cereals (wheat, corn, and rice) [2], tuber crops (potato, cassava, and yam) [3], legumes (bean and pea) [4] and green or immature fruits (banana, mango, and kiwifruit) [5], [6], [7]. Among them, raw kiwifruit, that is, during or before the commercial picking period is a new source of fruit-derived starch (40–60% on a dry basis) that has not been focused on [8]. It has been verified that kiwi starch (KS) not only has similar features to traditional starch but also has the particularity of starch derived from fruits or vegetables. It has a low pH value, a large amount of dietary fiber and phenolic substances, and a high content of resistant starch (RS), which can be used as a novel and healthy food formula and may have a positive impact on the kiwi industry [7], [8], [9], [10]. Nevertheless, native KS still has many shortcomings, such as low solubility in cold water, low clarity, low pseudoplasticity, and weak gel strength, which largely limits its application in food and non-food industries [7], [8], [9], [10]. In order to improve the performance of KS to meet the requirements of special processing unit operations and increase its added value, it is necessary to modify KS to enhance the positive attributes and minimize these drawbacks.

Up to now, more and more attention has been paid to the development and processing of modified starch. Diverse approaches such as chemical, enzymatic, or physical modifications are used to improve the features of native starch to expand its applications in the food industry. However, the chemical modification is often polluted and easily generates the residual, which has certain adverse effects on the human body. Enzymatic modification is easily restricted by temperature, pressure, pH, and salt ions, and it is hard to control the reaction process [11]. In contrast, ultrasonic modification is eco-friendly, highly efficient, and safe, and it is attaining more attention as a way of cleaner production and green food processing.

The physical effects (such as local pressure, temperature pulse, turbulence) and/or chemical effects of cavitation determines the results of ultrasonic modification [12], [13]. Generally, ultrasonic treatment (UST) with the density higher than 5 W/cm^2^ is called high-power ultrasound treatment (HUT), which has been gradually applied to starch modification [14]. Ding, Luo, & Lin (2019) treated retrograded starch (RS3) at 100–600 W for 30 min, and the results revealed that HUT increased its apparent amylose content (AAC), rapidly digestible starch (RDS) content, and slowly digestible starch (SDS) content, and reduced its RS content and enzyme resistance [15]. Wang, Xu, Ma, Liang, Zhang, & Chen (2020) treated sweet potato starch at 300 W for 15, 20, 25, and 30 min, and the results showed that HUT enhanced the disorder of the aggregation structure, increased the swelling power (SP) and solubility, and declined the pasting temperature and viscosity of the starch [16]. Karwasra, Kaur, & Gill (2020) treated wheat starch at 100 W for 15 and 30 min, and the results indicated that HUT significantly improved the SP, AAC, and oil absorption capacity (OAC), changed the composition and structure of the starch, and expanded the application of starch in different food processing industries [17]. As a new type of physical modification, UST or HUT will be more and more widely used.

Considering the positive effects of UST or HUT on physical modification, this study aimed to explore the effects of different ultrasonic powers (0, 200, 400, and 600 W) and ultrasonic times (0, 10, 20, and 30 min) on the structural, physicochemical and functional properties, and *in vitro* digestibility of KS. Additionally, it discussed the relationships between molecular structure and various properties of KS. The study focused on the green preparation of high-performance modified KS, accumulated relevant data for the wide application of ultrasound technology, and advanced starch technology and ultrasonic chemistry to a certain extent.

## 2 Materials and methods

### 2.1 Materials and chemicals

Pectinase (500 U/mg); cellulase (50 U/mg), amylose (potato source), amylopectin (corn source), 2,4,6-tripyridine-s-triazine (TPTZ),α-amylase (bacillus subtilis source), and glucose determination kit (glucose oxidase method) were all purchased from Shanghai yuanye Bio-Technology Co., Ltd. Porcine pancreatic α-amylase (23 U/mg); pancreatic lipase (57 U/mg); invertase (≥300 U/mg); glucosidase (8 × USP) were all purchased from Sigma-Aldrich Co. Ltd. (St. Louis, MO, USA).

### 2.2 Starch preparation

Huayou kiwifruits, 100 kg, with a fruit hardness of 90–100 N and soluble solid content of 6.5–8.0 Brix° were picked during the commercial picking period. Kiwifruits were peeled manually and the seeds were removed. 20% ice water (w/w) was added, and the pulp was obtained by a high-speed blender. The pH of the pulp was adjusted to 3.5–4, then, 0.2% pectinase was added and placed at 45–50 °C for 2 h. The precipitate was collected by vacuum filtration, washed 3 times with distilled water, and dissolved in water again. Then the pH was adjusted to 4.5–5.5, 0.2% cellulase was added, and it was placed at 45–50 °C for 2 h. The precipitate was collected as above, then dried in an oven at 30 °C. Finally, KS was milled, sieved, and collected (200 mesh). 3% NaOH solution was to adjust pH.

### 2.3 Ultrasonic treatment

According to Ding et al. (2019) [15], KS homogeneous slurries (5%, w/v, deionized water) were treated under five different ultrasonic conditions, including 200 W (8.42 W/cm^2^) for 30 min, 400 W (16.84 W/cm^2^) for 30 min, 600 W (25.27 W/cm^2^) for 30 min, 600 W (25.27 W/cm^2^) for 20 min, and 600 W (25.27 W/cm^2^) for 10 min. HUT were conducted using an ultrasonic probe processor (ATPIO-1000D, Nanjing Xianou Ltd., Jiangsu, China) with a probe (Ф=10 mm, 20–25 kHz) and a constant temperature water bath (XODC-0515-II, Nanjing Xianou).

### 2.4 Structural characterization

#### 2.4.1 Scanning electron microscopy (SEM)

KS granules were observed using a SEM (FlexSEM1000, Hitachi, Tokyo, Japan). All samples were observed at a magnification of × 1000 and × 3000.

#### 2.4.2 Polarizing microscope (PLM)

KS dispersion, 2% (w/v), was observed using a PLM (XPV-230E, Shanghai Changfang Optical Instrument Co., Ltd., China).

#### 2.4.3 Particle size distribution (PSD)

PSD was performed using an LS13320 laser particle size analyzer (Beckman Coulter, Inc., CA, USA). KS, 100 mg, was suspended in deionized water, dispersed by ultrasonic wave, and used to determine the PSD.

#### 2.4.4 X-ray diffraction (XRD)

XRD was performed by a Bruker D8 Advance A25 X-ray diffractive analyzer (Germany) [16]. The relative crystallinity (RC) was obtained using Jade 6.5 software (Materials Data, Inc., Livermore, California, USA). Each treatment had 3 samples, and each sample had three repeating measurements.

#### 2.4.5 Fourier transform infrared spectroscopy (FTIR)

An FTIR spectrometer (Vetex70, Bruker, Germany) was used to analyze the change in functional group. The KS sample was loaded on the ATR plate [16]. Each treatment had 3 samples, and each sample had three repeating measurements. Background corrections were performed and normalisation of spectra was performed.

### 2.5 Physicochemical properties

#### 2.5.1 SP and water solubility index (WSI)

The SP and WSI determination were based on the method by Zhang, Li, Wang, Yao, & Zhu (2017) with some changes [18]. A total of 100 mg KS (W_0_, DW) was dissolved in 10 mL deionized water and heated at 45 °C、55 °C、65 °C、75 °C、85 °C, and 95 °C for 30 min separately. During heating, the samples were oscillated for 5 s every 2 min. Then the samples were cooled to 25 °C immediately and centrifuged at 3000 × *g* for 30 min. The precipitate was precisely weighed (W_s_), and the supernatant was decanted and dried to a constant weight (W_1_). The values of WSI and SP were measured as followed:

(1) *WSI = W_1_/ W_0_ × 100%*

(2) *SP = W_s_ /(W_0_×(100% − WSI)) (g/g)*

#### 2.5.2 Oac

A 1 g (DW) KS was put into a 25 mL pre-weighed centrifuge tube, and 10 mL peanut oil (peanut oil, Luhua, China) was added to it, stirred for 10 min, stood for 30 min, centrifuged at 2200 × *g* for 30 min, and the liquid was poured out [17].

(3) *OAC = Weight of sample after oil absorption/ Weight of sample*

#### 2.5.3 Differential scanning calorimetry (DSC) and pasting properties

According to our previous study [7], the DSC determination was recorded by a Q2000 DSC (TA Instruments, Norwalk, USA). The onset temperature (To), peak temperature (Tp), conclusion temperature (Tc), and enthalpy change (ΔH) were recorded. The pasting properties were measured by a Rapid Visco-Analyzer (RVA-Tec Master, Perten Instruments, Sweden) [7]. The time to peak viscosity (Ptime), pasting temperature (PT), peak viscosity (PV), hot paste viscosity (HPV), cold paste viscosity (CPV), breakdown value (BD), and setback value (SB) were measured.

#### 2.5.4 Gel texture properties

The gel texture determination was assessed using a TA-XT plus texture analyzer (Stable Micro Systems Ltd., Godalming, UK). KS dispersions of 20% were heated at 95 °C in containers for 30 min with shaking and stored at 4 °C for 24 h to prepare a homogenous gel structure. A P/0.5R probe was used, the test distance was 5 mm, the probe speed during the whole test was 1 mm/s, the force induction was 5 g, and the interval between two tests was 5 s [7].

#### 2.5.5 Rheological properties

According to our previous study [7], static shear rheology properties and temperature scanning results were obtained by a rheometer (DHR-1, Waters, Massachusetts, USA) with a PP60 clamp. G' (storage modulus), and G'' (loss modulus) were recorded.

### 2.6 Functional properties

Starch content, apparent amylose content (AAC), the TPC determination and the antioxidant capacity (by DPPH method and ferric reducing antioxidant power (FRAP) methods) were all based on the methods by Wang et al. (2021b) [7]. The total polyphenol content (TPC) results are expressed as µg gallic acid equivalents/g (µg GAE/g), and the results of DPPH and FRAP were expressed as μM Trolox equivalents/g (μM TE/g).

### 2.7 *In vitro* digestibility

According to Wang et al. (2021b) [7], KS (1.2 g, DW) was dissolved in ultrapure water (8 mL) and stirred continuously in a boiling water bath for 30 min to obtain gelatinized KS. Then, 200 mg pancreatic lipase and 6 mL deionized water were stirred for 10 min to obtain the supernatant. Enzyme solution was made up of two parts, one was 2.5 mg invertase, 1 mL ultrapure water, and 1.5 mL supernatant, the other was 10 μL glucosidase and 1 mL ultrapure water. The 25 °C gelatinized starch, 10 mL of pH 5.2 sodium acetate buffer and the enzyme solution were mixed and put in a 37 °C shaking water bath (160–190 rpm/min) for the starch hydrolysis. At 0, 20, 40, 60, 90, 120, 150, and 180 min intervals, 0.5 mL samples were placed into 20 mL of 75% ethanol solution and then centrifuged at 8952 × *g* for 5 min. The supernatant was to measure the glucose content.

### 2.8 Statistical analysis

Analysis was performed using SPSS 23, and the data were expressed as the means ± standard deviations (SD). Each sample was prepared and analyzed in triplicate. One-way ANOVA, followed by the Duncan post hoc test, was used to determine the significant differences. *p* < 0.05 was considered statistically significant. Figures were mainly drawn by Excel 2016 and Origin 9.1. The correlation test was performed by OriginPro 2020 (OriginLab, USA).

## 3 Results and discussion

### 3.1 Structural characterization

#### 3.1.1 SEM and FLM

**Fig. 1:**
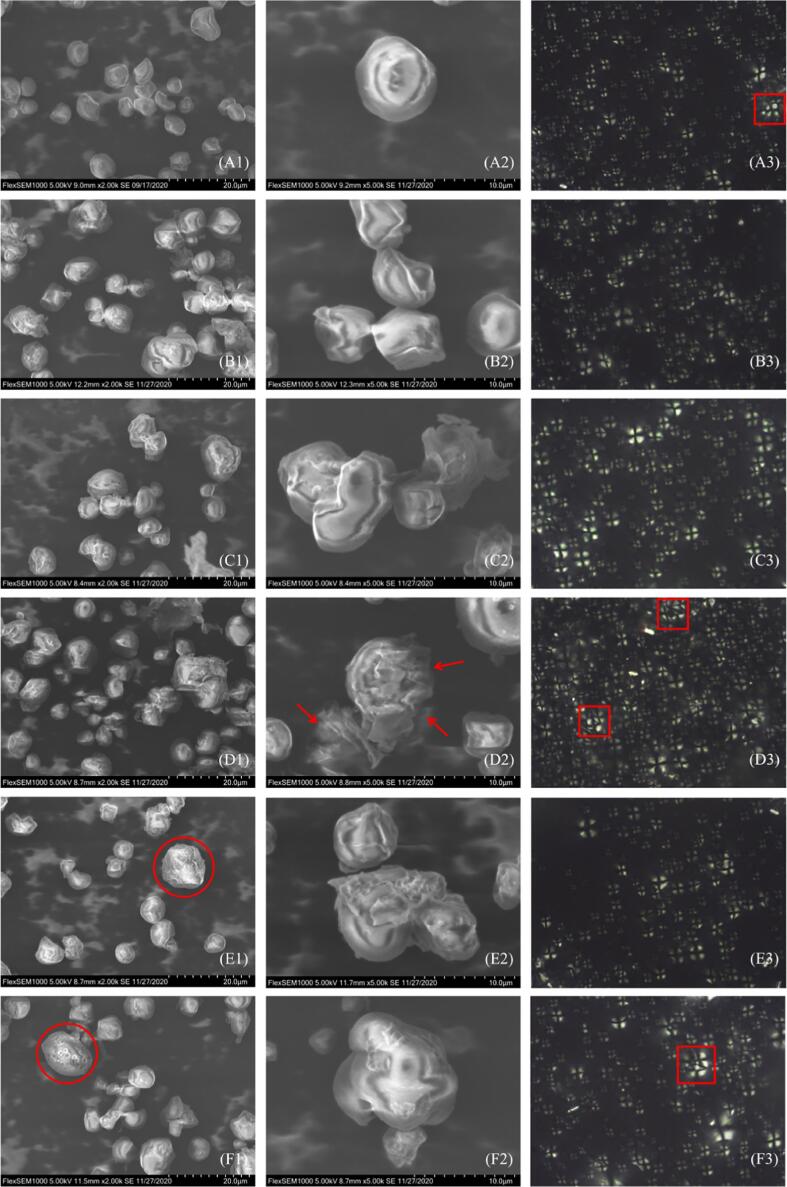
Structural characterization of KS. SEM image (2000 × ) (A1-F1), SEM image (5000×) (A2-F2), and PLM image (A3-F3) of CK(A), (200 W, 30 min) (B), (400 W, 30 min) (C), (600 W, 30 min) (D), (600 W, 20 min) (E), and (600 W, 10 min) (F).

Morphological characteristics of KS granules treated with HUT are shown in Fig. 1 A1-F1 (×2000) and Fig. 1 A2-F2 (×5000). The native KS was polygonal, elliptical, and round, and the surface was relatively smooth (Fig. 1 A1-A2). When the treatment time was 30 min, roughness and corrosion began to appear on the surface of KS after 200 W HUT (Fig. 1 B1-B2). With the increase in ultrasonic power to 400 W, there were obvious depressions and partial disintegration on the surface (Fig. 1 C1-C2), but complete particles could still be observed. However, 600 W HUT almost destroyed the original shape of the KS particles, and a large number of particles were fragmented (Fig. 1 D1-D2). At 600 W, the morphology of the starch granules became coarser, and the degree of corrosion and disintegration deepened with the increase in ultrasonic time.

The effects of ultrasound are mainly dependent on the physical effects and/or chemical effects of cavitation [12], [19], causing the rupture of the starch molecular chain, destroying the fine structure and internal molecules of the starch, thereby breaking the integrity and rigidity of the starch granules, and forming mechanical damage to the starch granules. In addition, a few microporous starches were observed in the SEM images of (600 W, 10 min) and (600 W, 20 min) (Fig. 1 E1-F1, as shown in the red circle), and large particles were more affected by HUT than small particles, which showed that the effect of HUT on starch particles was not uniform, resulting in the appearances of different particle morphologies.

Fig. 1 A3-F3 showed the PLM of KS after HUT. The shape of KS was irregular, and a more obvious polarized cross could be seen, located in the center of the granules. Some KS had more than one cross (as shown in the red square), which indicated that there might be semi-composite agglomerates in the KS and partial fusion phenomenon.

#### 3.1.2 Psd

**Fig. 2:**
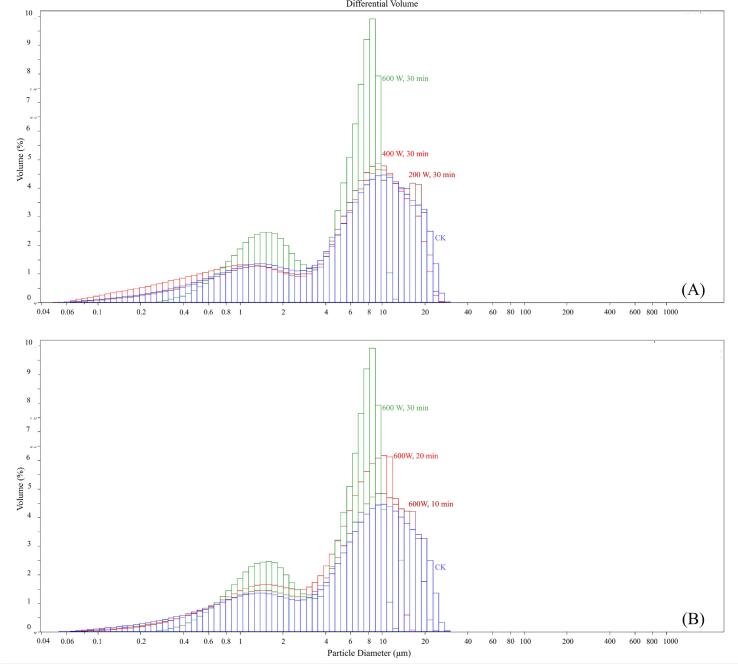
Particle size distribution of KS. (A) KS treated with different ultrasonic powers; (B) KS treated with different ultrasonic times.

The PSD curve and the average particle size results of KS after HUT were shown in Fig. 2 and Table 1, respectively. KS presented a bimodal starch grain distribution, with an average particle size ranging from 5.25 to 8.35 μm, and a single particle size ranging from 0.04 to 40 μm. The order of the average particle size of starch granules was as follows: CK > (200 W, 30 min) > (400 W, 30 min) > (600 W, 30 min), CK > (600 W, 10 min) > (600 W, 20 min) > (600 W, 30 min). In addition, KS particles showed a tendency to move to a smaller particle size after HUT, and the decreasing trend was more obvious with the increase in ultrasonic power and ultrasonic time, and the particle size of KS became more uniform after HUT (Fig. 2).

**Table 1:** Kiwi starch.

Types		CK	(200 W, 30 min)	(400 W, 30 min)	(600 W, 30 min)	CK	(600 W, 10 min)	(600 W, 20 min)	(600 W, 30 min)
AAC (%)		26.13 ± 0.21d	37.15 ± 0.34c	42.07 ± 0.98b	44.47 ± 0.21a	26.13 ± 0.21d	28.30 ± 0.55c	30.69 ± 0.49b	44.47 ± 0.21a
Average granule size (μm)		8.35 ± 0.24a	8.05 ± 0.18a	7.18 ± 0.28b	5.25 ± 0.04c	8.35 ± 0.24a	7.38 ± 0.07b	5.96 ± 0.03c	5.25 ± 0.04d
Relative crystallinity (%)		55.49 ± 0.62a	34.49 ± 0.29b	32.13 ± 1.03c	23.45 ± 0.70d	55.49 ± 0.62a	31.93 ± 0.40b	29.03 ± 0.13c	23.45 ± 0.70d
R_1047/1022_		1.0176 ± 0.0017a	1.0120 ± 0.0049b	1.0096 ± 0.0034c	1.0048 ± 0.0025d	1.0176 ± 0.0017a	1.0079 ± 0.0005b	1.0062 ± 0.0007bc	1.0048 ± 0.0025c
OAC (g/g)		1.52 ± 0.02d	1.63 ± 0.01c	1.73 ± 0.01b	1.84 ± 0.02a	1.52 ± 0.02d	1.73 ± 0.03c	1.99 ± 0.02a	1.84 ± 0.02b
Thermal properties	T_o_ (°C)	66.96 ± 0.01a	63.70 ± 0.11c	63.87 ± 0.06b	63.96 ± 0.06b	66.96 ± 0.01a	64.70 ± 0.04b	64.70 ± 0.19b	63.96 ± 0.06c
	T_p_ (°C)	70.40 ± 0.08a	67.10 ± 0.09d	67.34 ± 0.08c	67.51 ± 0.09b	70.40 ± 0.08a	68.22 ± 0.05b	68.23 ± 0.29b	67.51 ± 0.09c
	T_c_ (°C)	76.45 ± 0.18a	73.40 ± 0.20c	73.57 ± 0.12c	73.90 ± 0.10b	76.45 ± 0.18a	74.97 ± 0.15b	73.90 ± 0.10c	73.90 ± 0.10c
	Δ*H* (J/g)	11.13 ± 0.27b	11.49 ± 0.10ab	11.77 ± 0.39a	11.48 ± 0.31ab	11.13 ± 0.27a	11.51 ± 0.23a	11.20 ± 0.21a	11.48 ± 0.31a
Pasting properties	PV (Pa·s)	1533.33 ± 28.92d	2514.33 ± 29.48b	2749.33 ± 35.11a	2252.67 ± 16.74c	1533.33 ± 28.92d	2355.33 ± 6.81b	2650.67 ± 30.01a	2252.67 ± 16.74c
	HPV (Pa·s)	1295.00 ± 15.52c	1683.00 ± 8.89a	1766.33 ± 143.35a	1457.33 ± 2.31b	1295.00 ± 15.52d	2056.67 ± 2.08b	2222.00 ± 19.00a	1457.33 ± 2.31c
	BD (Pa·s)	216.33 ± 9.45d	830.00 ± 19.97b	911.00 ± 6.08a	782.00 ± 8.66c	216.33 ± 9.45d	299.67 ± 6.66c	419.00 ± 1.00b	782.00 ± 8.66a
	CPV (Pa·s)	2233.00 ± 12.53c	2716.33 ± 38.94a	2802.33 ± 134.54a	2490.00 ± 53.69b	2233.00 ± 12.53d	3470.67 ± 19.50b	3788.33 ± 27.54a	2490.00 ± 53.69c
	SB (Pa·s)	943.33 ± 10.41c	1029.67 ± 28.38b	1043.67 ± 3.21ab	1066.00 ± 1.73a	943.33 ± 10.41d	1411.67 ± 21.96b	1564.33 ± 8.74a	1066.00 ± 1.73c
	Ptime (min)	6.02 ± 0.04a	5.86 ± 0.01b	5.84 ± 0.04b	5.71 ± 0.03c	6.02 ± 0.04c	6.32 ± 0.02a	6.22 ± 0.03b	5.71 ± 0.03d
	PT (°C)	88.78 ± 0.03a	84.68 ± 0.08c	83.35 ± 0.09d	85.58 ± 0.03b	88.78 ± 0.03a	88.43 ± 0.35b	88.02 ± 0.04c	85.58 ± 0.03d
Gelatinization properties	Hardness (g)	216.13 ± 20.35c	380.63 ± 49.77b	365.42 ± 11.72b	441.69 ± 15.44a	216.13 ± 20.35d	361.95 ± 2.74c	408.46 ± 13.77b	441.69 ± 15.44a
	Springiness	1.00 ± 0.01ab	0.98 ± 0.01c	1.00 ± 0.01a	0.99 ± 0.01bc	1.00 ± 0.01a	1.01 ± 0.01a	0.99 ± 0.01a	0.99 ± 0.01a
	Cohesiveness	0.50 ± 0.04b	0.63 ± 0.01a	0.65 ± 0.01a	0.65 ± 0.01a	0.50 ± 0.04c	0.60 ± 0.02b	0.61 ± 0.01ab	0.65 ± 0.01a
	Gumminess (g)	110.62 ± 6.42c	252.35 ± 7.36a	225.56 ± 8.16ab	196.16 ± 8.28b	110.62 ± 6.42d	223.96 ± 3.53b	277.13 ± 1.26a	196.16 ± 8.28c
	Chewiness (g)	110.48 ± 6.63d	248.34 ± 24.90b	298.15 ± 13.28a	193.91 ± 19.85c	110.48 ± 6.63c	196.92 ± 20.28b	280.92 ± 14.56a	193.91 ± 19.85b
	Resilience	0.13 ± 0.01c	0.29 ± 0.01b	0.32 ± 0.01a	0.31 ± 0.01ab	0.13 ± 0.01d	0.22 ± 0.01c	0.26 ± 0.01b	0.31 ± 0.01a
TPC (μg GAE/g)	5079.53 ± 26.80a	4360.23 ± 53.60b	3681.87 ± 28.20c	3453.80 ± 13.40d	5079.53 ± 26.80a	3705.26 ± 8.77b	3532.75 ± 45.01c	3453.80 ± 13.40d	
Antioxidant activities	DPPH (μM TE/g)	7.74 ± 0.01a	5.84 ± 0.02b	5.23 ± 0.01c	4.40 ± 0.02d	7.74 ± 0.01a	5.42 ± 0.02b	4.91 ± 0.01c	4.40 ± 0.02d
	FRAP (μM TE/g)	38.13 ± 0.20a	22.97 ± 0.22b	22.37 ± 0.11c	18.78 ± 0.15d	38.13 ± 0.20a	26.25 ± 0.26b	24.78 ± 0.20c	18.78 ± 0.15d

It could be seen from Table 1 that with the increase in ultrasonic power and time, the average particle size of KS decreased from 8.35 to 5.25 μm. It might be attributable to the fact that HUT broke the large starch granules into small granules, and KS would not re-aggregate after being broken, or the low surface adhesion between the particles did not make the starch surface adhere to the particles. There are still controversies about the particle size of ultrasonic treated starch. Wang et al. (2020) and Ding et al. (2019) found that HUT increased the particle size of sweet potato starch and foxtail mill starch respectively [15], [16]. Gonçalves, Noreña, da Silveira, & Brandelli (2014) found that the size of the starch from Araucaria angustifolia seeds decreased after HUT [20], while Falsafi, Maghsoudlou, Rostamabadi, Rostamabadi, Hamedi, & Hosseini (2019) and Orsuwan & Sothornvit (2015) believed that HUT had no significant effect on the size of oat starch and banana starch granules [21], [22]. It may still be attributed to the difference in the properties of starch itself.

#### 3.1.3 Xrd

**Fig. 3:**
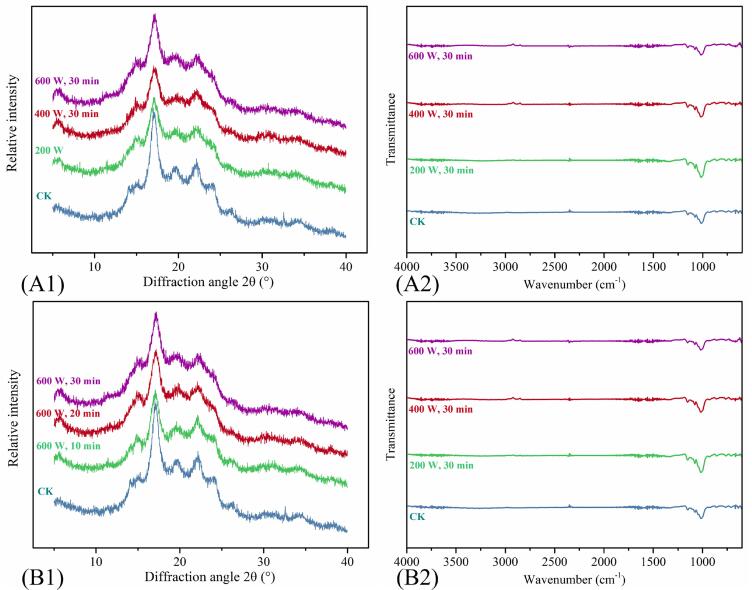
(A1) XRD and (B1) FTIR (ATR) spectra of KS treated with different ultrasonic powers; (A2) XRD and (B2) FTIR (ATR) spectra of KS treated with different ultrasonic times.

The crystal structure of starch granules generally uses XRD. XRD patterns of KS proved that native KS presented a typical B-type structure, and its main peaks were located at 15.1°, 17.1°, 22.2°, and 24°, but with the increase in ultrasonic power and the prolongation of ultrasonic time, KS gradually transformed to C-type (Fig. 3 A1and Fig. 3 B1).

As a natural polymer, the crystallization behavior of starch is usually complex. It can be seen from Table 1 that the crystallinity of KS was significantly different after different HUTs. The crystallinity of CK was 55.49%, and those of KS treated with different ultrasonic powers were (200 W, 30 min) 34.49%, (400 W, 30 min) 32.13%, and (600 W, 30 min) 23.45%, respectively. Those of KS treated with different ultrasonic times were (600 W, 10 min) 31.93%, (600 W, 20 min) 29.03%, and (600 W, 30 min) 23.45%, respectively. That is, the greater the ultrasonic power and the longer the ultrasonic time, the lower the crystallinity of KS. This finding implied that HUT damaged the surface and the inner-granule molecular structure by free radicals and cavitation, and HUT could decrease the RC [23].

#### 3.1.4 Ftir

The short-range ordered degree of KS can be reflected from the amplitude ratio of 1047/1022 cm^−1^ (R_1047/1022_), because the range of 1200–800 cm^−1^ of FTIR spectra were sensitive to changes in the short-range order structure [16]. The characteristic peaks remained basically unchanged (Fig. 3 A2-B2), which indicated the molecular structure and functional group were similar to native starch. R_1047/1022_ showed regular changes with the change in ultrasonic parameters. The R_1047/1022_ of CK was 1.0176, while that of KS decreased successively with the increase in ultrasonic power and time, and the R_1047/1022_ of (600 W, 30 min) was 1.0048 (Table 1). It showed that HUT did not change the basic spectral pattern of KS, did not generate new chemical groups, but reduced the short-range order of KS. This might be because HUT destroyed the amorphous and crystalline region of starch particles, resulting in irregular arrangement or expansion of the spiral structure, and its degree deepened with the increase of ultrasonic power and time. Wang et al. (2020) also found a similar attenuation of R_1047/1022_ in ultrasonic treated sweet potato starch [16].

### 3.2 Physicochemical properties

#### 3.2.1 WSI and SP

Studying the properties of the starch–water system is of great significance in the food industry. The WSI is mainly attributed to the overflow of amylose molecules from KS granules, and the SP reflects the characteristics of amylose. In addition, the WSI and SP of starch are also related to the size, shape, and molecular weight of starch molecules [24].

**Fig. 4:**
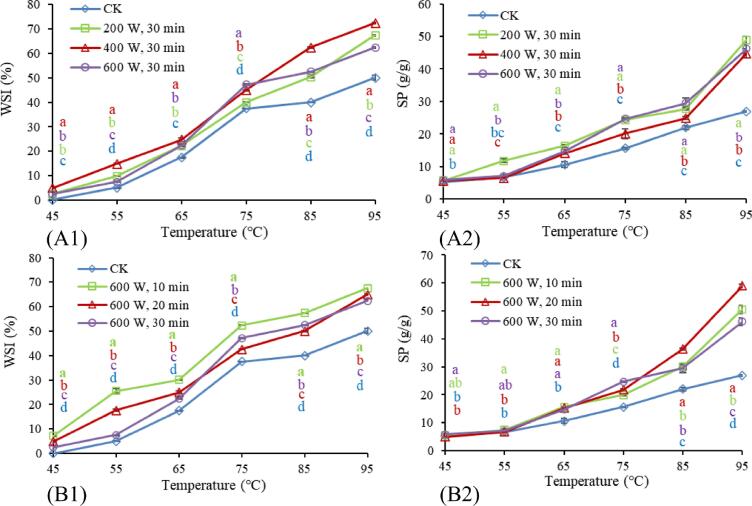
Water solubility index (WSI) and swelling power (SP) of KS. (A) KS treated with different ultrasonic powers; (B) KS treated with different ultrasonic times.

As shown in Fig. 4, the WSI and SP of KS increased within the temperature range of 45–95 °C, and the WSI and SP of HUT-modified KS were significantly higher than that of native KS (*p* < 0.05). Falsafi et al. (2019) also reported this phenomenon in oat starch [21]. However, the WSI and SP of modified KS at each temperature did not show a regular change with the increase in ultrasonic power and ultrasonic time, this may be because the HUT destroyed the amorphous part of KS, and different ultrasonic powers and times had different effects on the compactness of starch granules, and HUT would cause interactions between starch molecular chains and change the number of water-binding sites available. Moreover, the effective removal of the protein layer on the surface of KS granules by HUT and amylose leaching might also be the reason for the increased SP [21]. In addition, Fig. 4 showed that the slope of the WSI curve of all KS at 65–75 °C were significantly higher than other temperatures, mainly because KS is gelatinized at 65–75 °C, which made it easier for water molecules to enter the amorphous region of starch granules. However, when the temperature was too high, starch granules were destroyed, and their water absorption capacity was limited. Meanwhile, the SP of KS raised fastest between 85 and 95 °C, which might be because the high temperature destroyed the crystal molecular structure of KS, accelerating the speed of molecular movement, thus promoting the combination of water molecules with the free hydroxyl groups of amylose and amylopectin through hydrogen bonds, further aggravating the swelling of the samples.

#### 3.2.2 Oac

Oil is mainly stored in the starch network through physical embedding, and OAC is an effective way to detect the emulsification ability of starch [25] (Singh & Sharanagat, 2020). As shown in Table 1, the OAC of the CK group was 1.52 g/g. The OAC of the KS after different ultrasonic powers were (600 W, 30 min) 1.84 g/g, (400 W, 30 min) 1.73 g/g, and (200 W, 30 min) 1.63 g/g; the OAC of KS after different ultrasonic times were (600 W, 20 min) 1.99 g/g, (600 W, 30 min) 1.84 g/g, and (600 W, 10 min) 1.73 g/g, which indicated that HUT significantly improved the OAC of KS (*p* < 0.05), this was consistent with the conclusion obtained by Singh & Sharanagat in elephant foot yam starch [25]. Furthermore, at 30 min, the OAC of KS increased with the increase in the ultrasonic power due to the formation of crevices and small openings at the surface of KS granules, thus increasing the capillary attraction of oil molecules [21]. However, at the 600 W treatment, the OAC of KS increased first and then decreased with the increase in the ultrasonic time, this is mainly because excessively high ultrasonic intensity caused the rearrangement of broken starch crystallites, which covered the affinity sites of oil molecules and reduced the binding ability of oil [26]. The results revealed that appropriate ultrasonic modification can improve the emulsifying properties of KS, thereby enhancing the industrial benefits of KS in food formula.

#### 3.2.3 Dsc

DSC was used to measure the temperature parameters of KS after different HUT, including To, Tp, Tc, and ΔH, which were 63.70–66.96 °C, 67.10–70.40 °C, 73.40–76.45 °C and 11.13–11.77 J/g, respectively (Table 1). Compared with CK, the To, Tp and Tc values of KS were significantly reduced after different HUT (*p* < 0.05), all showed a trend of moving to low temperature. Wang et al. (2020) also found that ultrasonic treatment could move To, Tp, and Tc of sweet potato starch to low temperature [16]. The changes in the To, Tp, Tc values often reflect the change in crystal structure; HUT might destroy the ordered double helix structure of starch, reduce the number of crystals, thus reducing the gelatinization temperature of KS. However, ΔH was basically unchanged (*p* > 0.05), which was different from previous studies. For example, Wang et al. (2020) reported that ultrasonic power reduced the ΔH of sweet potato starch [16], and Xiao, Wu, Zhang, Luo, Lin, & Ding (2021) found that ultrasonic power reduced the ΔH of corn starch, potato starch, and pea starch [27]. Difficult change of the ΔH after HUT might be because the high proportion of amylose makes KS gather strongly in the ordered molecular structure [23].

#### 3.2.4 Pasting properties

The RVA gelatinization results of KS after different HUTs were shown in Table 1. The PV, HPV, BD, CPV, SB, Ptime, and PT of KS after different HUT were 1533.33–2749.33 Pa·s, 1295.00–2222.00 Pa·s, 216.33–911.00 Pa·s, 2233.00–3788.33 Pa·s, 943.33–1564.33 Pa·s, 5.71–6.32 min, and 83.35–88.78 °C. Compared with the CK, HUT significantly increased the PV, HPV, and CPV values of KS (*p* < 0.05), and with the increase in ultrasonic power and ultrasonic time, these values all first increased and then decreased, and reached the maximum at (400 W, 30 min) and (600 W, 20 min). Wang et al. (2021c) also observed a similar phenomenon in rice starch [28]. Amylopectin would be broken under high temperature and high pressure for a long time, which increased the possibility of contact and movement between starch molecules, thereby increasing the viscosity of starch. All these were due to HUT (Zhang et al., 2020). After that, the changes in crystallinity structure, the orders of internal molecular chain, and the other substances such as phenols might have participated in the descent process together [7], [21], [29]. Meanwhile, the BD value and SB value of HUT modified starch were significantly higher than native KS (*p* < 0.05). In addition, HUT significantly reduced the gelatinization temperature of KS (*p* < 0.05), which confirmed the conclusion drawn by DSC (Table 1). The cavitation produced by HUT severely destroyed the internal hydrogen bonds and intermolecular hydrogen bonds of the starch chain, resulting in free starch chains and a lower amylopectin content, water molecules can penetrate into starch granules more easily, so as to accelerate gelatinization and reduce gelatinization temperature [11], [30].

#### 3.2.5 Gel texture properties

TPA model was used. The hardness of 20% (w/v) KS gel was 216.13–441.69 g, the springiness was 0.98–1.00, the cohesiveness was 0.50–0.65, the gumminess was 110.62–277.13 g, the chewiness was 110.48–298.15 g, and the resilience was 0.13–0.32 (Table 1). Hardness refers to the force required for the starch gel deformation. Compared with potato starch and corn starch [31], KS had higher hardness, and the hardness showed a significant upward trend with the extension of the ultrasonic time and power (*p* < 0.05). Similar to the hardness, HUT significantly improved the gumminess and chewiness of starch gels (*p* < 0.05) (Table 1). All these indicated that HUT significantly enhanced the granular properties of KS. This may be due to the changes in the amylose content, starch purity, and the structure of amylose and amylopectin in KS after HUT [32]. The data of springiness, cohesiveness, and resilience showed that HUT significantly enhanced the internal tightness of the gel. Among them, the 400 W at 30 min group had the highest springiness, cohesiveness, and resilience, which might be attributed to phosphate esters in KS participating in the re-association of starch molecules through the formation of intermolecular cross-links, thus making the internal structure of the gel more compact [33].

#### 3.2.6 Rheological properties

**Fig. 5:**
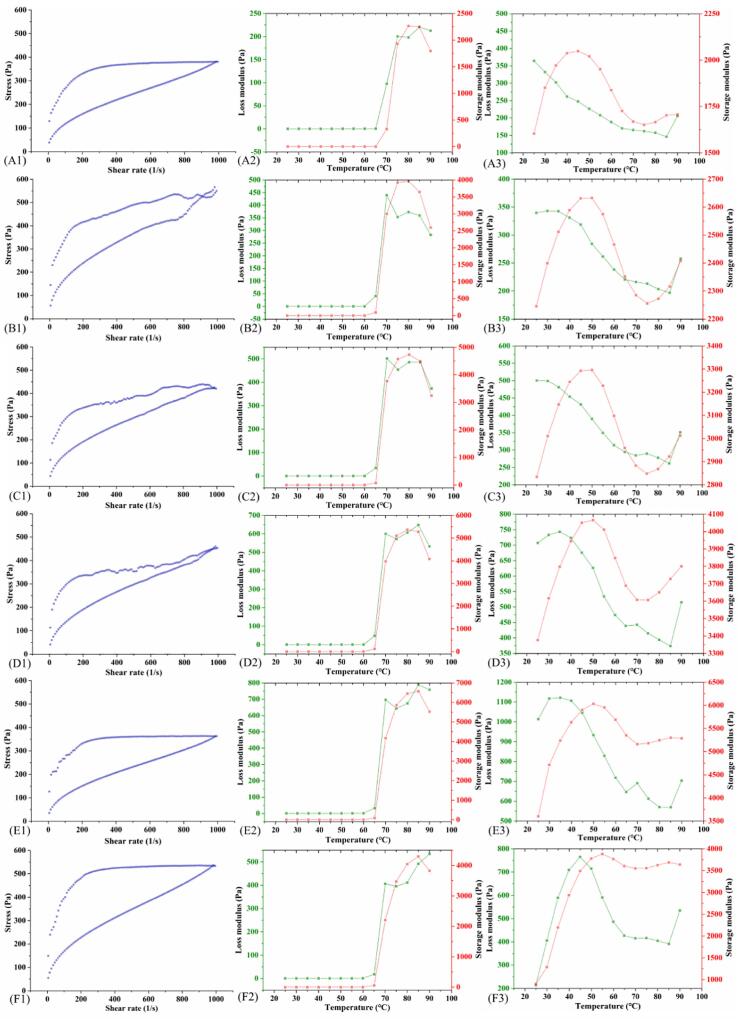
Rheological properties of KS with different ultrasonic treatments. (A1-F1) The hysteresis loops of KS; (A2-F2) Changes in G' and G'' of 20% KS suspensions during heating; (A3–F3) Changes in G' and G'' of 20% KS suspensions during cooling. CK(A), (200 W, 30 min) (B), (400 W, 30 min) (C), (600 W, 30 min) (D), (600 W, 20 min) (E), and (600 W, 10 min) (F). In A2-F3, red represents G' (storage modulus) and green represents G'' (loss modulus).

Thixotropy is one of the important rheological properties of water-soluble polymer solutions, and its influence on food taste is reflected in the refreshing and soft feeling. As shown in Fig. 5 A1-F1, the clockwise hour-hand lagging ring indicates that all samples were time-dependent and belonged to the thixotropic system. The thixotropic ring area of the 600 W at 10 min group increased significantly (Fig. 5 F1), indicating that HUT under this condition severely damaged the gel structure of KS and increased the energy used to eliminate the influence of time on flow behavior, and reduced the shear resistance of starch paste. Under the action of shear stress, all KS pastes showed pseudo-plasticity and shear thinning behavior, which could be well fitted to the power law equation (Table A1); the fluid index (n) and consistency coefficient (K) are empirical constants. The more the n value deviates from 1, the more easily the fluid shear thinning and the greater the pseudoplasticity; the higher the K value, the more viscous the fluid. In addition, thixotropy of KS after different HUTs did not show regular changes, which might be due to the different effects of HUT on the linear molecular chains in the system, intermolecular forces, and the unwinding between the molecules [30] (Table A1). In general, 200 W at 30 min had the largest K and the smallest n, which had better thixotropy during food processing.

A temperature scan was performed on the 20% starch paste. In the range of 25 °C–60 °C, G' and G'' were basically stable, then G' and G'' raised sharply and reached the maximum value after 60 °C. However, when the temperature continued to rise, G' and G'' had a downward trend. In addition, compared with the CK (Fig. 5 A2), the G' and G'' of the modified KS increased significantly (Fig. 5 B2-F2), indicating that HUT strengthened the viscoelastic characteristics of the starch gel, making the internal structure of the KS more compact and enhancing the energy recovery ability. The elastic properties still played a dominant role in the cooling process (Fig. 5 A3-F3). On the whole, the G' and G'' of KS showed an upward trend due to ultrasonic modification, but there was no dependence on power or time. All in all, the KS of all HUT groups showed similar changes in different rheological tests.

### 3.3 Functional properties

#### 3.3.1 AAC and TPC

Starch mainly includes amylose and amylopectin; the ratio of the two directly determines the structure of starch macromolecules, and further determines the different properties. Compared with CK (26.13%), the AAC of KS after HUT was significantly increased (*p* < 0.05) and increased with the increase in ultrasonic power and time, with the maximum increase of 70.19% (Table 1). This was consistent with the results of Babu, Mohan, & Parimalavalli in foxtail millet starch [34]. Zavareze & Dias (2011) showed that the increase in amylose might be due to the degradation of amylopectin after HUT [35]. In addition, HUT might affect the fluidity of the amorphous and crystalline regions of starch, leading to the organization and formation of amylose–amylose, amylose–amylopectin and amylopectin–amylopectin helical structures [36], resulting in an increase in AAC. The increase in AAC might also be attributed to the partial depolymerization of amylose and amylopectin by HUT.

Zhang et al. (2020) found that the antioxidant capacity of kiwifruit was mainly related to phenols [37]. All HUTs could significantly reduce the TPC in KS (*p* < 0.05), and there were significant differences between different ultrasonic powers and ultrasonic times (*p* < 0.05). Among them, 600 W at 30 min had the most significant effect, reducing the TPC from 5079.53 μg GAE/g to 3453.80 μg GAE/g. This might be due to the destruction of the starch granule structure caused by HUT, the release of soluble phenolic compounds, and the dissociation of bound polyphenols [38].

#### 3.3.2 Antioxidant activities

As shown in Table 1, the DPPH and FRAP of CK was 7.74 and 38.13 μM TE/g, they were significantly reduced after HUT (*p* < 0.05). The DPPH and FRAP decreased by 32.01% and 43.15% after HUT (600 W, 30 min). Therefore, on the one hand, HUT significantly weakened the antioxidant capacity of KS, which might be attributed to the release and reduction in antioxidant substances (mainly phenols) after HUT. On the other hand, it effectively improved the purity of KS and removed the other substances except starch.

### 3.4 *In vitro* digestibility

**Fig. 6:**
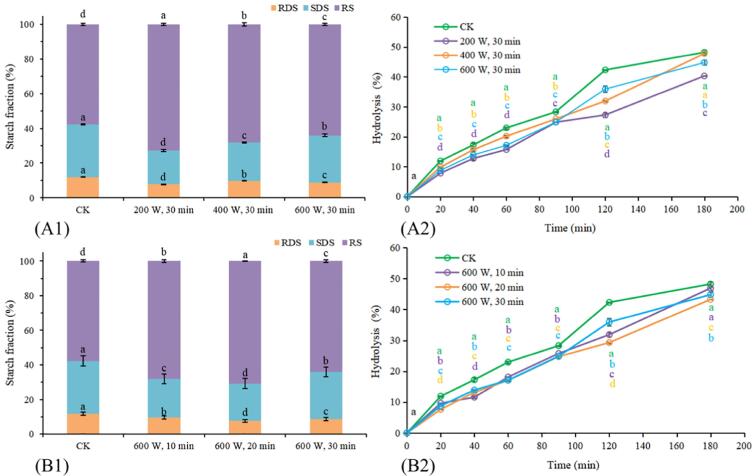
(A1) RDS, SDS, and RS content of KS and (B1) *in vitro* digestibility treated with different ultrasonic powers; (A2) RDS, SDS, and RS content of KS and (B2) *in vitro* digestibility treated with different ultrasonic times.

According to the digestion behavior under the action of enzymes, starch can be divided into three types: RDS, SDS and RS [39]. It can be seen from Fig. 6 A1-B1 and Table 2 that among all groups, the contents of RDS (11.88%) and SDS (30.42%) in the native KS were the highest, while the content of RS (57.69%) was the lowest. However, the content of RS in KS significantly increased and the content of RDS and SDS significantly reduced after different HUTs (*p* < 0.05). In particular, 200 W at 30 min increased the content of RS by 26.07%, up to 72.73%, while it decreased the content of RDS (7.78%) and SDS (19.49%) by 34.51% and 35.93%. These results indicated that HUT increased the enzymatic resistance of starch and increased the difficulty of starch digestion. However, with the increase of ultrasonic power and ultrasonic time, the digestion process and composition changes of KS did not show obvious regularity. It might be because HUT degrades KS molecules and partially destroys the double helix structure, causing the disorder of the starch structure, and the physical effect of ultrasound increases the internal temperature of KS, which leads to the reassembly of the starch chain to form a new helix. The newly formed helix may have a highly ordered structure that could lead to resistance to enzyme digestion, which is consistent with the results obtained by Ding et al. (2019) in retrograded starch [15].

**Table 2:** *In vitro* starch digestibility of starch samples.

	RDS (%)	SDS (%)	RS (%)
CK	11.88 ± 0.21a	30.42 ± 0.26a	57.69 ± 0.58d
(200 W, 30 min)	7.78 ± 0.02d	19.49 ± 0.74d	72.73 ± 0.59a
(400 W, 30 min)	9.81 ± 0.26b	22.10 ± 0.31c	68.09 ± 1.04b
(600 W, 30 min)	8.76 ± 0.14c	27.16 ± 0.82b	64.08 ± 0.68c
CK	11.88 ± 0.21a	30.42 ± 0.26a	57.69 ± 0.58d
(600 W, 10 min)	9.62 ± 0.11b	22.24 ± 0.58c	68.14 ± 0.84b
(600 W, 20 min)	7.64 ± 0.07d	21.66 ± 0.21d	70.70 ± 0.09a
(600 W, 30 min)	8.76 ± 0.14c	27.16 ± 0.82b	64.08 ± 0.68c

### 3.5 Correlation analysis of structure and properties of KS

**Fig. 7:**
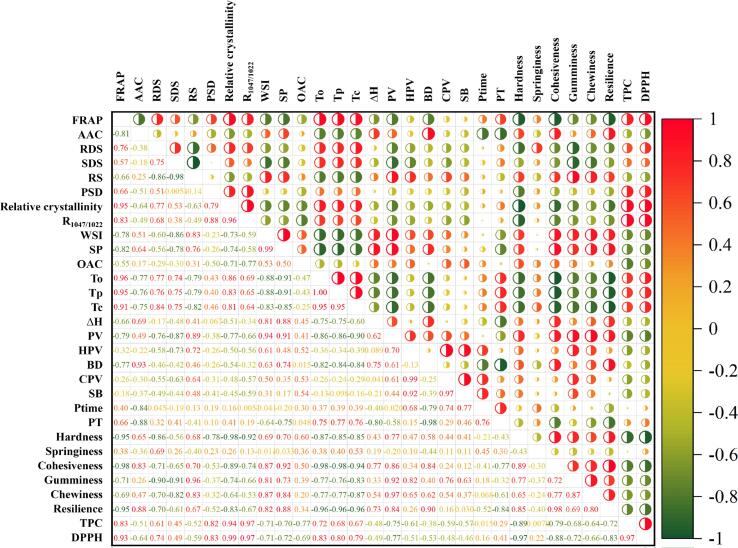
Correlation analysis of structure and properties of KS.

Plenty of studies have shown that some properties of starch are closely related to its structure. For example, the SP of starch granules is considered to be a characteristic of amylopectin [24], while the molecular structure of starch was significantly correlated with starch digestibility (Ding et al., 2019) [15]. At present, the research on the correlations between the structure of KS and its physicochemical, nutritional, and digestive properties are still at a blank stage. In this study, the existing data were analyzed, and the results are shown in Fig. 7. Positive correlations were shown in red and negative correlations in blue. The numbers correspond to the circles one by one, and the closer the value is to one, the darker the color, and the larger the circle, the stronger the positive/negative correlation is. The temperature parameters (To, Tp, and Tc) of KS had a positive correlation with the PT (R_To_ = 0.75, R_Tp_ = 0.77, and R_Tc_ = 0.76), indicating that different methods had consistency in the characterization of properties. The AAC of KS was positively correlated with BD (R = 0.93) and was negatively correlated with PT (R = −0.88), Ptime (R = −0.84), To, Tp, and Tc (R = −0.77, −0.76, and −0.75) but had almost no correlation with RDS (R = −0.38), SDS (R = −0.18), and RS (R = 0.25). It showed that AAC and particle size determined the gelatinization performance of starch to a certain extent, but had little effect on its digestibility. RC was positively correlated with R_1047/1022_ (R = 0.96), which jointly characterized the structural order of KS, and RC was positively correlated with To, Tp, and Tc (R = 0.86, 0.83, and 0.81), indicating that the higher the RC, the harder KS was to gelatinize. In addition, studies have reported that the antioxidant capacity of kiwifruit is mainly due to the phenolic compounds present in samples [40]. In this study, the antioxidant capacity of KS showed the highest correlation coefficient with the TPC (R_DPPH_ = 0.97, R_FRAP_ = 0.83). The increasing or decreasing antioxidant capacity might be attributable to the enhancing or reducing of phenolic components. In KS, TPC was positively correlated with many structural indexes such as R_1047/1022_ (R = 0.97), RC (R = 0.94), and PSD (R = 0.82), indicating that phenols greatly affected the structure of KS and then affected the functional properties of KS.

## 4 Conclusions

In this study, KS was modified by HUT for the first time. It was found that the native KS presented a typical B-type structure; with the increase in ultrasonic power and ultrasonic time, KS gradually transformed to C-type, and HUT destroyed the granular morphology of KS and formed holes and cracks on the surface of starch. In addition, HUT significantly reduced the particle size, the RC, and the short-range order of KS. In contrast, the WSI, SP, and OAC of HUT-modified KS were significantly higher than that of native KS. In terms of pasting properties and gel texture properties, HUT might destroy the ordered double helix structure of starch and reduce the number of crystals, thus reducing the gelatinization temperature of KS. HUT significantly enhanced the granular properties of KS. On the whole, G' and G' of KS had an upward trend due to ultrasonic modification, which showed obvious elastic characteristics. Meanwhile, HUT significantly increased the content of AAC and RS, while all HUT significantly reduced the TPC and antioxidant capacity of KS; however, it effectively improved the purity of KS. The research provides a theoretical basis for reasonably changing the function of KS and expanding its industrial application.

### CRediT authorship contribution statement

**Jiaqi Wang:** Conceptualization, Methodology, Software, Investigation, Data curation, Writing – original draft. **Xinran Lv:** . **Tian Lan:** Data curation, Writing – original draft. **Yushan Lei:** Visualization, Investigation, Supervision. **Jiangtao Suo:** Writing – review & editing, Writing – review & editing. **Qinyu Zhao:** Software, Validation. **Jing Lei:** Writing – review & editing, Writing – review & editing. **Xiangyu Sun:** Writing – review & editing, Writing – review & editing. **Tingting Ma:** Writing – review & editing, Writing – review & editing.

## Declaration of Competing Interest

The authors declare that they have no known competing financial interests or personal relationships that could have appeared to influence the work reported in this paper.

## Figures and Tables

**Table A1:** Fitting parameters of rheological equations.

Type	K (Pa·Sn)	n	R2
	Upward		
CK	111.71	0.1895	0.9035
(200 W, 30 min)	135.38	0.205	0.9671
(400 W, 30 min)	104.97	0.2109	0.9611
(600 W, 30 min)	115.96	0.1896	0.9195
(600 W, 20 min)	130.06	0.1603	0.8626
(600 W, 10 min)	158.59	0.1902	0.8466
	Downward		
CK	14.405	0.4602	0.9908
(200 W, 30 min)	22.878	0.448	0.9914
(400 W, 30 min)	17.128	0.4606	0.995
(600 W, 30 min)	16.054	0.4724	0.9947
(600 W, 20 min)	13.271	0.4645	0.9914
(600 W, 10 min)	21.3	0.4538	0.991
